# A middle aged women with bilateral staghorn complete calculi

**DOI:** 10.11604/pamj.2014.17.299.2555

**Published:** 2014-04-20

**Authors:** Jihad El Anzaoui, Driss Touiti

**Affiliations:** 1 Marrakech Military Hospital, Marrakech, Morroco

**Keywords:** Staghorn, calculi, urinary infection

## Image in medicine

A 50-year-old woman presented with a 2-years history of chronic flanc and hematuria. Plain abdominal radiography was performed showing bilateral complete staghorn calculi. Urinalysis and urine culture revealed a urinary infection by Proteus Morganii. Biologic investigations did not show any matabolic disorder. The urinary infection was treated by antibiotics and the stone by a combination of percutaneous nephrolitotomy and open surgery. The infrared spectrometry analysis found a calcium oxalate calculi. A Staghorn calculus is a large renal stone with irregular branches that can involve the renal pelvis and extend into calyces. Physiopatholgy of staghorn calculi is based on urinary infection. They are usually seen in the setting of infection with urease producing bacteria. They are composed of struvite (magnesium ammonium phosphate) but also, they can be composed of calcium oxalate. The majority of staghorn calculi are symptomatic: urinary infection, flanc pain, hematuria, urinary symptoms. The diagnosis can be performed by Plain abdominal radiography. Intravenous urography or CT scans are more helpful by showing the stone and delineating the pelvic calyceal anatomy. The treatment recommended of this type of renal stone is based on complete removal of all stone material after Sterilization of the urine with antimicrobial treatment. The elimination of all residual fragments can be obtained by the combinaison of different techniques: percutaneous nephrolitotomy, extracorporeal shockwave lithotripsy, retrograde ureteroscopic lithotripsy, open surgery. No treated, staghorn stone can cause septicemia, chronic renal insufficiency, destruction of kidney by infection and chronic calicial dilatation.

**Figure 1:**
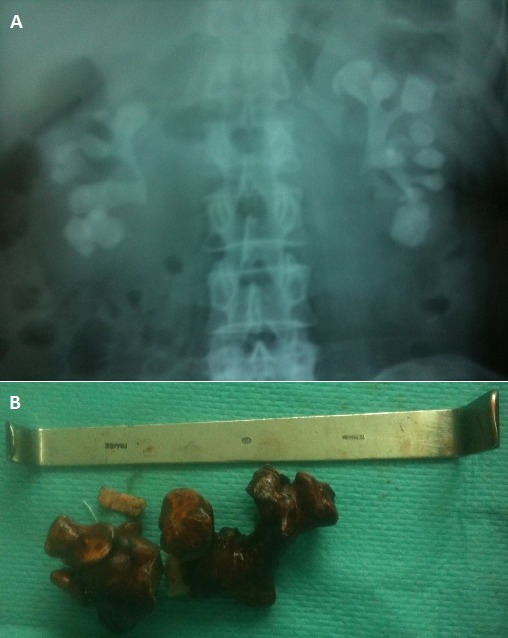
A) Plain abdominal radiograph: bilateral complete staghorn calculi; B) A part of staghorn stone extract by open surgery

